# Explaining Handwashing Behavior in a Sample of College Students during COVID-19 Pandemic Using the Multi-Theory Model (MTM) of Health Behavior Change: A Single Institutional Cross-Sectional Survey

**DOI:** 10.3390/healthcare9010055

**Published:** 2021-01-06

**Authors:** Manoj Sharma, Kavita Batra, Robert E. Davis, Amanda H. Wilkerson

**Affiliations:** 1 Department of Environmental and Occupational Health, University of Nevada, Las Vegas, NV 89119, USA; manoj.sharma@unlv.edu; 2 Office of Research, School of Medicine, University of Nevada, Las Vegas, NV 89102, USA; 3 Substance Use and Mental Health Laboratory, Department of Health, Human Performance and Recreation, University of Arkansas, Fayetteville, AR 72701, USA; red007@uark.edu; 4 Human Environmental Sciences, University of Alabama, Tuscaloosa, AL 35487, USA; awilkerson@ches.ua.edu

**Keywords:** multi-theory model, behavior change, COVID-19, pandemic, handwashing, young adults, college students

## Abstract

Amidst the COVID-19 pandemic, handwashing offers a simple and effective hygienic measure for disease prevention. Reportedly, a significant proportion of college students did not follow handwashing recommendations provided by the Centers for Disease Control and Prevention (CDC) in the pre-COVID era. The purpose of this cross-sectional study was to explore and explain the handwashing behavior among college students during the COVID-19 pandemic using a contemporary fourth-generation multi-theory model (MTM) of health behavior change. Data were collected from 713 college students at a large public university in the Southern U.S. in October 2020 using a validated 36-item survey. Statistical analyses included independent samples t-tests, Pearson correlation, and hierarchical regression modeling. Among students not following handwashing recommendations, the constructs of participatory dialogue (β = 0.152; *p* < 0.05) and behavioral confidence (β = 0.474; *p* < 0.0001) were statistically significant and accounted for 27.2% of the variance in the likelihood of initiation of the behavior. Additionally, the constructs of emotional transformation (β = 0.330; *p* < 0.0001), practice for change (β = 0.296; *p* < 0.0001), and changes in the social environment (β = 0.180; *p* < 0.05) were statistically significant and accounted for 45.1% of the variance in the likelihood of sustaining handwashing behavior. This study highlights the applicability and usability of the MTM in designing and testing behavior change interventions and media messaging in campaigns targeting college students.

## 1. Introduction

The novel coronavirus (COVID-19) disease caused by the SARS-CoV-2 virus has rapidly spiraled across the world and took the course of pandemic [1,2]. As of 18 November 2020, there are a total of 55,064,128 confirmed cases and 1,328,015 deaths attributable to COVID-19 worldwide, with a sizable proportion reported in the United States alone [2,3]. To reduce the spread of COVID-19, public health officials have encouraged various safety and mitigation strategies, including hygiene measures, such as handwashing, sneezing into an elbow, avoiding touching surfaces, and wearing personal protective equipment [1].

One of the simple yet effective measures recommended by the government and public health agencies is frequent and thorough handwashing [4,5]. Handwashing is a longstanding, non-pharmacologic public health measure, which has proven to minimize the spread of gastrointestinal and respiratory infections by 31% and 21%, respectively [6,7]. Handwashing removes microorganisms from the hands and prevents their transfer through different media, such as foods, beverages, and inanimate objects [8]. The CDC recommends scrubbing hands thoroughly with soap and water for at least 20 s under running water and drying hands with a clean towel or air drying for effective hand cleaning [4,8]. The CDC encourages public to wash their hands at a minimum during the following circumstances: (1) after being in public; (2) before and after providing care for someone who is sick; (3) after blowing their nose, sneezing, or coughing; (4) before, during, and after food preparation; (5) before eating food; (6) after using the toilet; (7) after touching an animal; and (8) after touching garbage [4,8].

College campuses and their surrounding communities became a central concern for COVID-19 prevention due to surging trends of COVID-19 among young adults aged 20–29 in the U.S. since June 2020 [9,10]. The CDC and American College Health Association published guidelines and considerations for reopening campuses amid the pandemic, including recommendations to emphasize hand hygiene practices on campus [8,11]. Research in the pre-COVID-19 era reported a lack of adherence or compliance to the handwashing regimen among college student and university community samples [12,13,14]. The rate of compliance varied from 38.5% to 66.9%, which underscores the need for interventions to promote handwashing behavior in this group [12,13,14]. Previous theory-based interventions successfully promoted good handwashing practices among young adults [15,16,17]. However, more research is needed to reinforce and sustain these handwashing practices among college students at the time of COVID-19 crisis, while the development of pharmacologic interventions is underway.

Recent studies reported that female students and those with the positive attitudes and knowledge towards handwashing for COVID-19 prevention were more likely to adopt handwashing behavior [18,19]. Further, studies utilized the theory of planned behavior, health action process approach, and health belief model to explain handwashing behavior in college students and adults during the COVID-19 pandemic [20]. Positive attitudes, positive subjective norms about handwashing behavior, higher perceived benefits of handwashing, and lower barriers to handwashing were associated with adherence to proper handwashing [20]. Additional information on factors related to initiation and continuation of the handwashing behavior to prevent COVID-19 spread is needed to ensure greater behavior adoption rates among college students.

The current study uses a contemporary, fourth-generation theoretical framework, the multi-theory model (MTM) for health behavior change, to explore handwashing behavior among college students. The MTM is a health behavior change theory with the unique ability to explain factors related to one-time (e.g., initiation) and long-term (e.g., sustenance) engagement in health behavior [21]. The constructs are adaptable across health behaviors and have been used to explain a variety of health behaviors among college students [22,23,24,25,26,27]). For initiation, the MTM posits that participatory dialogue (i.e., advantages counterweighing disadvantages of handwashing), behavioral confidence (i.e., a sureness in properly following steps of thorough handwashing despite obstacles), and changes in the physical environment (i.e., availability and accessibility of necessary resources for handwashing) will be instrumental in behavior change [21,28,29,30]. Whereas, for sustenance, emotional transformation (i.e., converting feelings into goals for regular handwashing), practice for change (i.e., creating a habit of handwashing and making it a way of life), and changes in the social environment (i.e., fostering social support from the environment) are important for long-term adherence to the changed behaviors [21,30,31].

Previous health behavior theoretical models focused on behavior acquisition rather than change have yielded mixed results when exploring behavior change, are documented to lack substantive predictive power, and are not suited to determine long-term behavior change [21]. The MTM is a unique theoretical framework due to identifying antecedents for both initiation and sustenance of behavior. Identifying factors related to the initiation and sustenance of handwashing using the MTM can inform the development of handwashing interventions for college students in the short and long term. Therefore, the study aimed to explain handwashing behavior during the COVID-19 pandemic among college students using the MTM. The findings of this study can be utilized to develop effective health promotion interventions to encourage handwashing behavior for this target audience.

## 2. Materials and Methods

### 2.1. Study Design and Study Participants

This descriptive, quantitative, cross-sectional survey study was conducted from 8 October 2020, to 29 October 2020, among college students at a large public Southern US university. The survey was deemed the most appropriate design due to its inherent capacity to collect information with relative ease from large population groups within a given period [32]. Students currently enrolled in any university programs, who were aged 18 years or above, had internet access, could provide informed consent, and comprehend English, were invited to participate in this survey. Alumni and those with no Internet access and an inability to understand English were excluded from this study.

### 2.2. Ethical Considerations

This study followed the Checklist for Reporting Results of Internet E-Surveys (CHERRIES) guidelines [33]. This study was deemed exempt from the university’s institutional review board (Protocol # 2009281493) on 21 September 2020. All participants were requested to provide their informed consent by answering an agree/disagree question, thereby confirming their willingness to participate. Informed consent included detailed information related to the study’s aim and significance and informed participants of their right to withdraw at any point. Participants who selected the “agree” option were directed to complete an online questionnaire. Personal identifiers, including names, email I.D.s, and details of COVID-19 exposure, were not collected. Only one response per I.P. address was allowed.

### 2.3. Recruitment and Data Collection

The convenience sampling method was used for sample recruitment. Participants were recruited through advertisements posted on the university’s daily e-news bulletin, intended to disseminate information to faculty, students, and staff with a valid university email address. The recruitment advertisement containing an anonymous survey link with complete details of the study was sent out every Thursday for three consecutive weeks to all currently enrolled students (27,562). Upon clicking the link, interested participants were connected to the Qualtrics (Provo, UT) interface to access and fill out the web-based survey. As an incentive, participants were informed that by participating, they had the opportunity to enter a drawing to win one of five $20.00 Walmart gift cards. The final survey item asked students about their willingness to enter the drawing to receive one of the gift cards. Interested students clicked ‘yes’, which functioned as a link to a separate survey where an email address could be collected for contact purposes. This process preserved participant anonymity by making it impossible to link the provided email address to data previously disclosed during the data collection process. The Qualtrics survey options were set to prevent “ballot box stuffing.” This option restricts the same IP address from contributing in multiple surveys. Participants who accessed the survey link more than once would receive a message that their responses had already been recorded.

### 2.4. Survey Instrument

Handwashing encompasses five steps from wetting the hands with running water, applying lather by rubbing soap, scrubbing hands thoroughly for at least 20 s, rinsing hands under running water, followed by drying with a clean towel or air drying them [34]. Utilizing the MTM theoretical framework, a survey instrument to assess the likelihood of initiating and sustaining handwashing behavior was developed. The instrument consisted of 36 items with six items related to participant demography. The remaining 30 items correspond to two main components of the MTM, such as initiation and sustenance, with a total of seven constructs. In the initiation, the first construct is participatory dialogue measuring advantages (five items) and disadvantages (five items) of handwashing behavior on a 5-point scale from never (0) to very often (5). The possible score ranges of advantages and disadvantages were 0–20, with a high score on advantages and a low score on disadvantages depicting the likelihood of the behavior change. The score of the participatory dialogue was computed after deducting the disadvantages score from the advantages score. The score range of participatory dialogue varies from −24 to +24 units [35]. The second construct of initiation was behavioral confidence, which implies confidence in making the behavior change with external or internal driving sources [35]. Behavioral confidence measured the likelihood of initiation of behavior change with six items and a score ranging from 0 to 24 [35]. Another construct of initiation was “changes in the physical environment,” which encompasses alterations related to the accessibility of resources or factors to initiate behavior change [35]. The physical environment change was assessed on three items, and the score ranges from 0–12 units.

The second element of MTM: sustenance has three constructs, namely emotional transformation (converting emotions to the goals), practice for change (self-assessment, overcoming barriers, focused on consistency with the behavior change), and changes in the social environment (support from the social circle for the behavior change). These constructs were measured using three items, each with a score range of 0–12 units [35]. The instrument used clear and appropriate language, which corresponds to the Flesch reading ease of 69.6 and Flesch–Kincaid Grade Level of fifth grade [36]. A panel of six subject matter, instrumentation, and target group experts assessed the instrument’s face and content validity and provided feedback. The instrument was advanced to the second round after addressing the feedback (16 modifications were made) before the finalization of the tool.

### 2.5. Statistical Analyses

Participants’ responses from Qualtrics^XM^ were exported to Microsoft Excel (Microsoft Corporation, Richmond VA, USA) and then imported to IBM SPSS version 26.0 (IBM Corp. Armonk NY, USA). Confirmatory factor analysis using the maximum likelihood method and internal consistency diagnostic tests using Cronbach’s alpha were performed to check the construct validity and internal consistency of the tool. For establishing a one-factor solution following the Kaiser criterion of Eigenvalue greater than or equal to 1.0, factor loadings on each item greater than 0.326 (after doubling the critical value for a sample size of 250 at an α = 0.01 for a two-tailed test) were established a priori as per the generally accepted recommendations from previous literature [36,37,38]. For establishing internal consistency reliability, a Cronbach’s alpha of ≥0.70 was considered acceptable [36]. Descriptive statistics, including the frequencies, proportions, mean, and standard deviations, were generated. The likelihood of intention and sustenance of handwashing behavior were the dependent variables, while the constructs were used as independent variables. To analyze the differences in mean scores across different groups who follow or did not follow handwashing recommendations, an independent-samples t-test was utilized. Pearson’s correlations test was utilized to calculate correlations among the variables. *p*-values less than 0.05 (two-sided) were considered statistically significant, and data were reported with 95% confidence intervals. Hierarchical multiple regression was utilized to predict the likelihood of initiation and sustenance of handwashing behavior based on multiple regressors or independent variables, such as age, gender, race/ethnicity, and individual constructs of the MTM. Gender and race/ethnicity variables were dummy coded. Assumptions of independence of observations (i.e., Durbin–Watson statistic), linearity (e.g., scatterplot and partial regression plots), homoscedasticity, multicollinearity, and normality (1.e., P-P plot and Q-Q plot) were evaluated on the full model.

### 2.6. Sample Size Justification

G*power software (version 3.1) was used to perform a priori power analysis [39]. The a priori power analysis was conducted to ascertain the required sample size for a test with a predetermined alpha and beta (power) level. For multiple regression, the alpha was set at 0.05, power at 0.80, five predictors (three MTM constructs and three covariates), and the effect size at 0.06 (small to medium), and this yielded a sample size of 234. After factoring in 10% incomplete entries or missing values, our minimum sample requirement was 257 (234 + 23 = 257).

## 3. Results

### 3.1. Sample Characteristics

A total of 713 valid responses were recorded during the survey period. The demographic profile of the respondent’s shows that 501 (70.3%) respondents identified as females and 176 (24.7%) were males (Table 1). The average age of the sample was 24.61 years (SD = 8.60 years). Nearly three-fourths of the participants were white (74.3%, *n* = 503) (Table 1). Most of the participants were undergraduate students (67.7%, *n* = 483), with nearly one-fourth (*n* = 197) being enrolled in the graduate program (Table 1). Further, 68.6% of students reported washing their hands at least six times over the past day (Table 1).

### 3.2. Characteristics of Study Variables and Inferential Statistics

Construct validation revealed a one-factor solution for each subscale and Eigenvalues greater than 1. All factor loadings were over the critical value of 0.326. The Cronbach’s alpha for all subscales was at least 0.70 (lowest = 0.70; highest = 0.96; Table 2) and were deemed acceptable [36].

There were significant differences in mean scores of initiation and sustenance across groups following or not following handwashing recommendations (Table 2). The mean initiation score was higher among those following handwashing recommendations (M = 3.40, SD = 0.88) than those not following (M = 2.3, SD = 1.14), with a statistically significant mean difference, M = 1.1, 95% CI [1.01, 1.29], *p* < 0.0001, Table 2). Similarly, the mean score of sustenance was higher (M = 3.35, SD = 0.90) following handwashing recommendations than those not following (M = 2.16, SD = 1.13), with a statistically significant mean difference, M = 1.2, 95% CI [1.01, 1.4], *p* < 0.0001, Table 2). The likelihood of initiation and sustenance was significantly correlated with all the constructs, at 0.01 levels of significance, for both groups (Table 3).

Hierarchical multiple regression was run to determine if the sequential addition of participatory dialogue, changes in the physical environment, and behavioral confidence improved the likelihood of initiation over and above demographic variables (Table 4). Among participants following handwashing recommendations, the full model (Model 4) to predict initiation was statistically significant, *R*^2^ = 0.479, *F* (6, 406) = 62.157, *p* < 0.0001; adjusted *R*^2^ = 0.471 (Table 4). The addition of participatory dialogue to the prediction of initiation (Model 2) led to a statistically significant increase in *R*^2^ of 0.240, *F* (1, 408) = 130.32, *p* < 0.0001. The addition of behavioral confidence to the prediction of initiation (Model 3) led to a statistically significant increase in *R*^2^ of 0.222, *F* (1407) = 170.97, *p* < 0.0001.

In the hierarchical regression with sustenance as the dependent variable, the full model (Model 4) was statistically significant, *R*^2^ = 0.434, *F* (6, 415) = 52.953, *p* < 0.0001; adjusted *R*^2^ = 0.425 (Table 4). The addition of emotional transformation to the prediction of sustenance (Model 2) led to a statistically significant increase in *R*^2^ of 0.372, *F* (1, 417) = 255.710, *p* < 0.0001. The addition of practice for change to the prediction of sustenance (Model 3) led to a statistically significant increase in *R*^2^ of 0.038, *F* (1, 416) = 27.413, *p* < 0.0001.

Among participants not following handwashing recommendations, the full model (Model 4) to predict initiation was statistically significant, *R*^2^ = 0.295, *F* (6, 181) = 12.615, *p* < 0.0001; adjusted *R*^2^ = 0.272 (Table 5). The addition of behavior confidence to the prediction of initiation (Model 3) led to a statistically significant increase in *R*^2^ of 0.162, *F* (1, 182) = 41.784, *p* < 0.0001 (Table 5). The addition of participatory dialogue to the prediction of initiation (Model 2) also led to a statistically significant increase in *R*^2^ of 0.117, *F* (1, 183) = 24.682, *p* < 0.0001. In the hierarchical regression with sustenance as a dependent variable, the full model (Model 4) was statistically significant, *R*^2^ = 0.468, *F* (6184) = 26.972, *p* < 0.0001; adjusted *R*^2^ = 0.451 (Table 5).The addition of emotional transformation to the prediction of sustenance (Model 2) led to a statistically significant increase in *R*^2^ of 0.344, *F* (1, 186) = 100.790, *p* < 0.0001. The addition of practice for change to the prediction of sustenance (Model 3) led to a statistically significant increase in *R*^2^ of 0.080, *F* (1, 185) = 26.772, *p* < 0.0001. The addition of changes in the social environment to the prediction of sustenance (Model 4) led to a statistically significant increase in *R*^2^ of 0.023, F (1, 184) = 7.957, *p* < 0.05.

## 4. Discussion

The study utilized the MTM, a novel fourth-generation behavioral theory, to explain handwashing behavior among college students during the COVID-19 pandemic. Despite the aggressive media messages and recommendations from governmental authorities, the study revealed that 31.4% of the college students did not practice frequent handwashing in the recommended manner compared to 33.1% to 61.5% reported in pre-COVID-19 era [12,13,14]. This study shows that the proportion of young adults not following the handwashing guidelines remains a matter of concern in these times. This finding points to the urgent need to design individual-level behavior change educational interventions and community-level media campaigns directed specifically toward this subgroup to mitigate the COVID-19 transmission.

This study revealed that approximately 27.2% of the variance in the likelihood to practice initiation was significantly predicted by behavioral confidence (i.e., a sureness in properly following steps of thorough handwashing despite barriers) and participatory dialogue (i.e., assessing advantages and disadvantages of handwashing), among those not following the handwashing recommendations (*p* < 0.0001). Participatory dialogue will be higher in the magnitude if advantages outweigh disadvantages and will be lower in the reverse scenario. This can be validated by the participatory dialogue score among students not following the handwashing recommendations, who perceive handwashing having more disadvantages than advantages. Every unit increase in behavioral confidence resulted in a 0.474 unit increase in the likelihood of the initiation of handwashing behavior. Similarly, for every unit increase in participatory dialogue, a 0.152 increase in the likelihood of the initiation of handwashing behavior ensued. Among students following the handwashing recommendations (*n* = 489, Table 4), all three constructs of initiation are statistically significant and account for approximately 47.1% of the variance in the response variable. The magnitude of these associations, as represented by adjusted *R*^2^, is considered substantial in behavioral and social science research [36]. Similar to behavioral confidence, a construct of self-efficacy has adequately been used to reinforce handwashing behavior among general populations [40,41,42,43]. The statistical significance of participatory dialogue and behavioral confidence has already been established in studying other behaviors (intake of sweetened beverages, binge drinking, portion size consumption, fruits, and vegetable consumption, and intentional outdoor nature contact) among college students [22,23,24,25,26,27,44,45].

Further, it is worth noting that significant differences between all three initiation constructs in the MTM for initiation were statistically significant (*p* < 0.0001) and greater among those who were practicing the recommendations for handwashing compared to those who were not. This finding lends support to the predictability of MTM for the initiation of handwashing behavior among college students. Future research and individual-level behavior change interventions and media campaigns should utilize the constructs of participatory dialogue and behavioral confidence in promoting handwashing behavior among college students.

Regarding the MTM’s ability to predict maintaining handwashing behavior, the examination of the sustenance model among those not following the recommendations revealed 45.1% of the variance in the likelihood to continue the practice of handwashing. Additionally, sustenance significantly predicted by all three constructs: emotional transformation (i.e., converting feelings into goals of regular handwashing) (*p* < 0.0001), practice for change (i.e., creating a habit of handwashing and making it a way of life) (*p* < 0.0001), and changes in the social environment (i.e., fostering social support from the environment) (*p* < 0.05). For every unit increase in emotional transformation, there is a 0.33 unit increase in the likelihood of maintaining handwashing behavior; for every unit increase in practice for change construct, there is a 0.296 increase in the likelihood of maintaining handwashing behavior; and for every unit increase in the changes in the social environment score, there is a 0.180 increase in the likelihood of maintaining handwashing behavior. The findings are also supported by data of students practicing handwashing according to recommendations (*n* = 489, Table 4), in which emotional transformation and practice for change are statistically significant and account for approximately 42.5% of the variance in the likelihood of sustaining handwashing behavior. Furthermore, the differences between all the three constructs of MTM for sustenance were statistically significant (*p* < 0.0001) and higher among those who were practicing the recommendations for handwashing as compared to those who were not, which further lends support to the predictive potential of MTM for the sustenance of handwashing behavior among college students.

Emotional transformation (i.e., converting feelings into goals) plays an important role in the sustenance of a behavior [21,35,46,47,48,49]. The construct of emotional transformation has also been found to be a statistically significant construct in other studies done among college students with other behaviors [22,24,26,27,44,45]. Individual-level behavior change interventions and media campaigns for college students must be designed to appeal to their emotions and get them toward concrete goals of handwashing behavior. Among studies conducted with non-college student populations, there is limited evidence of changes in the social environment or social support about maintaining handwashing behavior [41,50]. However, similar to previous constructs, the construct of changes in the social environment also derives backing from studies with other behaviors, such as sleep, eating, and drinking behaviors that have been done among college students [22,24,26,27,44,45]. Therefore, the importance of these constructs to the maintenance of handwashing behavior is also justified.

### 4.1. Implications for Practice

The study has important implications for designing handwashing promotion interventions, especially during the COVID-19 pandemic. The findings point to the fact that, despite the pandemic mounting at alarming rates, a substantial proportion of college students do not wash their hands as per the CDC recommendations. This issue can be addressed by designing individual-level behavioral change educational interventions and media campaigns (i.e., including social media) directed toward college students. Individual-level behavior change interventions can be delivered through classrooms or other university channels using the learning management systems (LMS) such as Blackboard, Canvas, Moodle, Brightspace, which almost all universities utilize to access course content. Further, as a policy measure supportive of behavior change, such interventions can be mandated by the university administration.

MTM has been used in designing similar technology-based brief and specific interventions [51,52,53] and can be effectively used for handwashing promotion among college students. The construct of participatory dialogue, which is quite intuitive and points to the underscoring the advantages of handwashing over any possible disadvantages, can be built by effective tailored messaging. Messages such as, “Handwashing is cool,” “Handwashing makes hands smell good,” and “Handwashing makes you attractive” can be used on both an individual level as well as media campaigns. Social media sites such as Facebook, Instagram, WhatsApp, and Twitter can be mobilized to promote these messages. Behavioral confidence can be built by demonstrating handwashing through videos that show the handwashing process in small steps. Additionally, potential barriers can be addressed by tailored messages such as making time to wash hands, having reminder messages, and overcoming inconvenience. These messages can be incorporated into the public health educational campaigns along with the standard guidelines of the regulatory agencies. Physical barriers, such as readily available water supply and soap, did not emerge as a significant factor in this U.S. sample but may be necessary for resource-constrained low and middle-income countries for which appropriate policy measures should be considered.

Concerning sustenance constructs, emotional transformation emerged as the most influential construct in this study, and the importance of channeling one’s feelings cannot be overemphasized. To build this construct, the first step is to be cognizant of one’s feelings, which is vital in changing handwashing behavior and changing any behavior and self-improvement [31]. After identifying feelings, especially negative ones, individuals should be directed toward establishing goals. In the case of handwashing promotion interventions, the goal should be to wash hands frequently in the recommended manner until it becomes second nature. The construct of practice for change can be built by teaching college students to self-reflect and self-monitor their handwashing behavior. Finally, the support of family, friends, peers, instructors, health professionals, and other significant influences must be emphasized in handwashing promotion interventions.

### 4.2. Strengths and Limitations of the Study

To our knowledge, this study is among the very few theory-based or evidence-based studies that have been performed on handwashing behavior among college students during the COVID-19 pandemic. This study offers a unique perspective by utilizing newer multiple theory models for studying handwashing behavior among college students. Among the few preventive measures that we have available to combat this pandemic, frequent and adequate handwashing seems to be an effective approach that a substantial number of college students are not practicing. Hence, this study provides direction for designing efficacious, brief educational interventions to promote handwashing. The scale utilized was psychometrically robust and met the acceptable criteria for validity and reliability and can be used for future cross-sectional and interventional studies.

However, this study has some limitations, which merit discussion. First, this study is based on only one large, public Southern U.S. university. Therefore, the findings may not be extrapolated to students of other institutions, and caution should be applied while interpreting the results. However, our sample was nearly representative of the institution where study took place. University racial breakdown for previous year was White/Caucasian (73.7%), Black/African American (4.4%), Hispanic (8.6%), and Asian (2.5%) (available at https://oir.uark.edu/quickfacts/factbook-2019-2020.pdf.

Second, the study relied on self-reported information, which can subject to measurement error. However, when it comes to measuring attitudes towards health behavior, this is the only method for the measurement. Third, in testing the instrument’s reliability, the instrument’s stability over time was not assessed. This offers a potential avenue for future research and will be especially important before conducting experimental studies. Fourth, actual availability of resources for handwashing were not measured in this study. Finally, the study used a cross-sectional design in which the independent and dependent variables are measured simultaneously, thereby preventing any causal inferences.

## 5. Conclusions

Amid the COVID-19 pandemic, this is a timely study that identifies a newer fourth-generation behavior change theory, MTM, to address handwashing behavior among college students. This study found that a substantial number of college students are not following handwashing recommendations. Further, the study provided evidence that MTM can help promote handwashing behavior among college students. There is a need to design and test individual-level behavior change interventions and media campaigns based on MTM for efficacy and effectiveness to change handwashing behavior among college students.

## Figures and Tables

**Table 1:** Descriptive statistics of the demographic variables of the study population (*n* = 713).

Variable	Characteristics	Mean ± S.D.	*n* (%)
Age	-	24.61 ± 8.60	-
Gender	Female	-	501 (70.3)
	Male	-	176 (24.7)
	Others	-	14 (2.0)
	Not reported	-	22 (3.1)
Race/ethnicity	White/Caucasian	-	530 (74.3)
	Black or African	-	33 (4.6)
	Hispanic or Latino	-	56 (7.9)
	Asian American	-	39 (5.4)
	Others	-	31 (4.4)
	Not reported	-	24 (3.4)
Current year in school	First-year undergraduate	-	164 (23.0)
	Second-year undergraduate	-	95 (13.3)
	Third-year undergraduate	-	90 (12.6)
	Fourth-year undergraduate	-	104 (14.6)
	Fifth-year undergraduate	-	30 (4.2)
	Graduate	-	195 (27.3)
	Professional degree	-	11 (1.5)
	Not reported	-	24 (3.4)
Employment	Yes	-	373 (52.3)
	No	-	318 (44.6)
	Not reported	-	22 (3.1)
Number of hours worked weekly *	-	23.0 ± 12.3	
Hand washing at least six times in the past day	Yes	-	489 (68.6)
	No	-	224 (31.4)

* Numbers of hours were reported by 340 (47.7%) participants only.

**Table 2:** Descriptive statistics of multi-theory model constructs of behavior change (*n* = 713) and reliability diagnostics.

Groups	Those Who Followed Handwashing Recommendations (*n* = 489)				Those Who Did Not Follow Handwashing Recommendations (*n* = 224)				
Constructs	Possible score range	Observed score range	Mean ± S.D.	Cronbach’s alpha	Possible score range	Observed score range	Mean ± S.D.	Cronbach’s alpha	*p*-value
Initiation	0–4	0–4	3.40 ± 0.88	-	0–4	0–4	2.3 ± 1.14	-	<0.0001
Participatory dialogue: advantages	0–20	0–20	16.40 ± 4.85	0.96	0–20	0–20	15.24 ± 5.35	0.96	0.004
Participatory dialogue: disadvantages	0–20	0–20	4.35±3.73	0.81	0–20	0–17	7.21 ± 4.13	0.74	<0.0001
Participatory dialogue: advantages—disadvantages score	−20–20	−20–20	12.75 ± 6.25	-	−20–+20	−13–+20	9.13 ± 6.14	-	<0.0001
Behavioral confidence	0–24	4–24	20.12 ± 4.41	0.90	0–24	0–24	15.9 ± 5.3	0.87	<0.0001
Changes in the physical environment	0–12	0–12	10.52 ± 2.1	0.83	0–12	2–12	9.7 ± 2.52	0.78	<0.0001
Entire initiation scale	-	-	-	0.72	-	-	-	0.69	-
Sustenance	0–4	0–4	3.35 ± 0.90	-	0–4	0–4	2.16 ± 1.13	-	<0.0001
Emotional transformation	0–12	1–12	9.85 ± 2.5	0.88	0–12	0–12	7.34 ± 3.04	0.87	<0.0001
Practice for change	0–12	0–12	7.96 ± 2.75	0.70	0–12	0–12	5.4 ± 2.73	0.76	<0.0001
Changes in the social environment	0–12	0–12	7.35 ± 3.74	0.87	0–12	0–12	4.43 ± 3.82	0.85	<0.0001
Entire sustenance scale	-	-	-	0.88	-	-	-	0.87	-
Entire scale	-	-	-	0.86	-	-	-	0.85	-

**Table 3:** Correlation matrix of the initiation and sustenance model constructs.

Construct(s)					
	**Those who followed handwashing recommendations (*n* = 489)**	**1**	**2**	**3**	**4**
1	Likelihood for Initiation	-	0.358 **	0.671 **	0.515 **
2	Participatory dialogue: advantages—disadvantages score		-	0.464 **	0.365 **
3	Behavioral confidence	-	-	-	0.631 **
4	Changes in the physical environment	-	-	-	-
1	Likelihood for Sustenance	-	0.625 **	0.550 **	0.378 **
2	Emotional Transformation	-	-	0.651 **	0.418 **
3	Practice for Change	-	-	-	0.565 **
4	Changes in the Social Environment	-	-	-	-
	**Those who did not follow handwashing recommendations (*n* = 224)**	**1**	**2**	**3**	**4**
1	Likelihood for Initiation	-	0.361 **	0.520 **	0.284 **
2	Participatory dialogue: advantages—disadvantages score		-	0.469 **	0.483 **
3	Behavioral confidence	-	-	-	0.537 **
4	Changes in the physical environment	-	-	-	-
1	Likelihood for Sustenance	-	0.602 **	0.604 **	0.476 **
2	Emotional Transformation	-	-	0.643 **	0.423 **
3	Practice for Change	-	-	-	0.516 **
4	Changes in the Social Environment	-	-	-	-

** *p* values are significant <0.01.

**Table 4:** Hierarchical multiple regression predicting likelihood for initiation and sustenance of handwashing behavior among respondents following recommendations (n = 489)**.**

Variables	Model 1		Model 2		Model 3		Model 4	
	B	β	B	β	B	β	B	β
**The likelihood for initiation as a dependent variable**								
Constant	3.334 **		2.386 **		0.874 **		0.629 **	
Age	0.001	0.008	0.001	0.008	−0.006	−0.057	−0.005	−0.050
Gender	−0.141	−0.080	−0.004	−0.002	0.027	0.015	0.027	0.015
Race/Ethnicity	0.106	0.054	0.132	0.066	0.056	0.028	0.045	0.023
Participatory dialogue			0.070 **	0.496	0.022 **	0.159	0.019 *	0.137
Behavioral confidence					0.116 **	0.585	0.104 **	0.524
Changes in the physical environment							0.049*	0.116
*R* ^2^	0.009		0.249		0.471		0.479	
F	1.256		33.819 **		72.520 **		62.157 **	
**Δ** *R* ^2^	0.009		0.240		0.222		0.008	
**Δ**F^2^	1.256		130.318 **		170.971 **		5.940 *	
**The likelihood for sustenance as a dependent variable**								
Constant	3.231 **		1.282 **		1.256 **		1.254 **	
Age	0.007	0.071	−0.003	−0.026	−0.006	−0.058	−0.007	−0.062
Gender	−0.240 *	−0.133	−0.061	−0.034	−0.036	−0.020	−0.034	−0.019
Race/ethnicity	−0.003	−0.002	−0.044	−0.022	−0.052	−0.026	−0.051	−0.026
Emotional transformation			0.222 **	0.624	0.164 **	0.461	0.162 **	0.456
Practice for change					0.085 **	0.259	0.073 **	0.224
Changes in the social environment							0.017	0.070
*R* ^2^	0.020		0.393		0.430		0.434	
F	2.901 *		67.430 **		62.843 **		52.953 **	
**Δ** *R* ^2^	0.020		0.372		0.038		0.003	
**Δ**F^2^	2.901 *		255.71 **		27.41 **		2.	

B (Unstandardized coefficient); β (Standardized coefficient), * *p*-value < 0.05; ** *p*-value < 0.001; Adjusted *R*^2^ of initiation = 0.471; Adjusted *R*^2^ of sustenance = 0.425.

**Table 5:** Hierarchical multiple regression predicting likelihood for initiation and sustenance of handwashing behavior among respondents not following recommendations (n = 224).

Variables	Model 1		Model 2		Model 3		Model 4	
	B	β	B	β	B	β	B	β
**The likelihood for initiation as a dependent variable**								
Constant	2.563 **		1.852 **		0.684 *		0.771*	
Age	−0.005	−0.042	−0.003	−0.022	0.000	−0.003	−0.001	−0.004
Gender	−0.234	−0.028	−0.057	−0.026	−0.015	−0.007	−0.014	−0.007
Race/ethnicity	−0.075	−0.028	−0.069	−0.026	−0.206	−0.078	−0.197	−0.074
Participatory dialogue			0.065 **	0.352	0.026 **	0.142	0.028 *	0.152
Behavioral confidence					0.099 **	0.461	0.102 **	0.474
Changes in the physical environment							−0.016	−0.035
*R* ^2^	0.015		0.132		0.294		0.295	
F	0.931		6.959 **		15.164 **		12.615 **	
**Δ** *R* ^2^	0.015		0.117		0.162		0.001	
**Δ**F^2^	0.931		24.682 **		41.784 **		0.202	
**The likelihood for sustenance as a dependent variable**								
Constant	2.533 **		0.700 *		0.525		0.481	
Age	−0.007	−0.060	−0.003	−0.027	−0.003	−0.021	−0.001	−0.011
Gender	−0.246	−0.113	−0.033	−0.015	−0.047	−0.022	−0.059	−0.027
Race/ethnicity	−0.128	−0.049	−0.086	−0.033	−0.098	−0.037	−0.064	−0.024
Emotional transformation			0.222 **	0.597	0.133 **	0.358	0.122 **	0.330
Practice for change					0.154 **	0.370	0.123 **	0.296
Changes in the social environment							0.053 *	0.180
*R* ^2^	0.020		0.365		0.445		0.468	
F	1.293		26.685 **		29.660 **		26.972 **	
**Δ** *R* ^2^	0.020		0.344		0.080		0.023	
**Δ**F^2^	1.293		100.790 **		26.772 **		7.957 *	

B (Unstandardized coefficient); β (Standardized coefficient), * *p*-value < 0.05; ** *p*-value < 0.01; Adjusted *R*^2^ of initiation = 0.272; Adjusted *R*^2^ of sustenance = 0.451.

## Data Availability

The data presented in this study are available on request from the corresponding author. The data are not publicly available due to ethical reasons.
